# In Memoriam: Dr. Sergey V. Zhirov (1966–2017)

**DOI:** 10.3897/CompCytogen.v13i3.47366

**Published:** 2019-10-21

**Authors:** Valentina Kuznetsova, Natalia Golub, Ninel Petrova, Vladimir Lukhtanov, Boris Anokhin, Natalia Khabasova, Nazar Shapoval, Larissa Kupriyanova, Ilya Gavrilov-Zimin

**Affiliations:** 1 Department of Karyosystematics, Zoological Institute, Russian Academy of Sciences, Universitetskaya nab. 1, 199034 St. Petersburg, Russia Zoological Institute, Russian Academy of Sciences St. Petersburg Russia

## Abstract

No

*On October 26^th^, 2017, the Cytogenetic Science suffered a stunning loss: Dr. Sergey V. Zhirov, geneticist and cytogeneticist, passed away at the age of fifty-one.*

**Figure:**
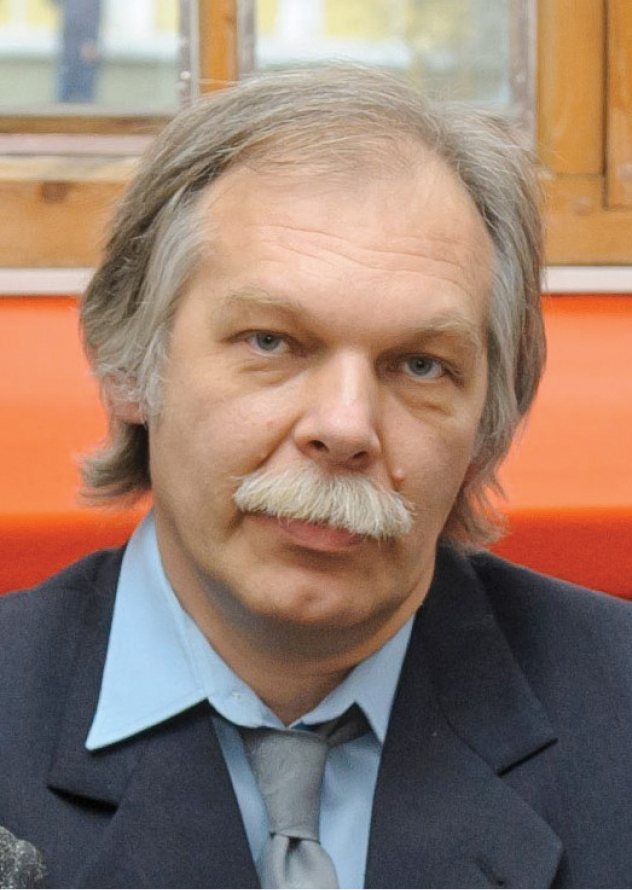
Dr. Sergey V. Zhirov (8.VI.1966 – 26.X.2017).

Dr. Sergey V. Zhirov, the researcher of the Department of Karyosystematics of the Zoological Institute, Russian Academy of Sciences in St. Petersburg, died of a heart attack, on his way to the Institute. This was a sudden death, and we were shocked to learn it.

Sergey was born on June 8^th^, 1966 in the city of Starodub, Bryansk region, Russia, as a son of a schoolteacher and an engineer. His childhood passed in an ancient Russian city of Pskov, where Sergey graduated from a secondary school with a Gold Medal. At school, he enthusiastically studied biology, chemistry, physics, and mathematics. However, Sergey finally chose biology and, after graduating from the school, he entered the Pskov State Pedagogical Institute, the Faculty of Biology and Chemistry, and the Department of Natural Geography. After the first course, he was called up for the military service. He was doing this service at the Finnish border until 1986 and then he returned to his alma mater. Sergey’s friends and tutors from the Institute recall him as a very capable, conscientious and active student. He participated in various activities of the student scientific society, carrying out research projects and participating in student conferences. His Graduate Thesis entitled “The influence of electromagnetic fields on the embryogenesis of the Chudsky lake whitefish” represented an exemplary student work.

After graduating, Sergey remained at the Department of Zoology of the Institute. Being an associate professor, he organized and conducted field training of the students and supervised their research work. He wrote several manuals and used to teach at the Ecological and Biological Center. Simultaneously with the teaching activity, Sergey continued his scientific studies.

In 1989, Sergey married his classmate Diana, and in 1990, their daughter Uljana was born.

Sergey lived with a passion for science. His distinguished research career focused on studying giant polytene chromosomes of chironomid midges (Diptera, Chironomidae), including polymorphisms related to the environmental conditions. In 1991-1994, Sergey was pursuing postgraduate studies at the Herzen State Pedagogical University (St. Petersburg) and the Zoological Institute RAS. He received a PhD degree in Genetics at St. Petersburg State University with the thesis “Chromosomal and genomic polymorphism in chironomid populations of the Pskov region”.

From 2008, Sergey continued his research in cytogenetics and karyosystematics of chironomids at the Department of Karyosystematics of the Zoological Institute RAS. Sergey was a talented researcher. He put forward interesting hypotheses and carefully and precisely conducted experiments to prove those hypotheses. Sergey had “golden hands” and created accurate and reliable research tools for his experiments. He published more than 20 articles in several peer-reviewed journals.

Among Sergey’s many distinctions, we would like to note another two. He was a member of the Editorial Team of *Comparative Cytogenetics*. As a Subject Editor managing submissions on chironomid cytogenetics, he was strict but very helpful and friendly to the authors. In addition, he was a highly respected guide at the Zoological Museum of the Zoological Institute. He took his guide mission very seriously and was able to explain complex issues in a perfectly clear way. Sergey was therefore one of the most admired guides at the Museum.

Apart from Sergey’s teaching and academic careers, he also was a talented person. He enjoyed literature and wrote a number of verses.

We were very fortunate to work and study together with Sergey. He will be sincerely missed by his friends and colleagues. Unfortunately, his wife outlasted Sergey for only two years; Diana passed away in October 2019. We send our heartfelt condolences to their daughter Uljana.
